# Supplementary material for: Navigating the leaky pipeline: Do stereotypes about parents predict career outcomes in academia?
Source: PLoS One. 2022 Oct 5;17(10):e0275670. doi: 10.1371/journal.pone.0275670 (PMC9534419; doi:10.1371/journal.pone.0275670)
Supplement: Attachment — Submitted filename: Response to reviewers.docx [file pone.0275670.s002.docx]

August 20^th^, 2022

Kristina Hood, Ph.D.

Virginia Commonwealth University

Academic Editor, *Plos ONE*

Dear Dr Hood:

We would like to thank you and the Reviewers for the insightful and helpful reviews of our manuscript *“Navigating the leaky pipeline: Do stereotypes about parents predict career outcomes in academia?”* (PONE-D-22-00288). We appreciate the constructive guidance toward ways in which we can strengthen this manuscript.

In our revision, we focused on and addressed the point that Reviewer 3 identified in the Decision Letter (Reviewer 2 was happy with the manuscript and did not offer any suggestions). We believe that these changes have further improved the manuscript, and we hope that you agree with this assessment.

In what follows, we discuss how we addressed the concern brought up by Reviewer 3 and have included the Reviewer’s comment, followed by our response and changes.

Again, we thank you and the reviewers for the very helpful feedback concerning the previous manuscript and hope that you will agree that the paper is ready to be published in Plos ONE. This manuscript is not currently under review elsewhere and has not been previously published either in whole or in part. All research participants were treated in accordance with APA ethical standards.

Thank you for your consideration.

Best regards,

The authors

***Reviewer 3, Point 1****. Thank you for the opportunity to review this manuscript again. It is evident that the authors have taken great care and put much effort into making suggested edits and revisions. However, I still have concerns about the power in Study 2 – it is mentioned, but not fully addressed in the manuscript.*

*It is appreciated that the authors included power analyses and effect sizes for their studies – this was much needed and helpful for evaluating this manuscript!*

*For Study 2, power = .61 is considered very low, and there is concern about how the lack of power affects interpretation of results. While it’s a great start to note that perhaps a larger sample size is needed, for this manuscript to be ready for publication, the low power found in Study 2 needs to be addressed head on. The authors might consider including some discussion of how low power might impact interpretation of findings, as well as including the low power in the limitation section in conjunction with discussion of the sample size. I don’t believe the low power to be irredeemable, but acknowledgement of it must be made, and the authors should justify why these results have merit despite this low power.*

We thank Reviewer 3 for bringing our attention to this issue and for their helpful suggestions. We have followed Reviewer 3’s suggestion and included a paragraph in the limitations section of the General Discussion (page 32 line 747-754) in which we addressed the low power found in Study 2, discussed how low power might impact the interpretation of the findings and included a suggestion for future research to replicate these findings with a larger sample size. We additionally included a brief discussion of the power in Study 2 in the discussion of the findings on page 28 lines 654-657 and a justification of the merit of the findings despite the low power.

“The small sample size and, consequently, low power in Study 2 is another potential limitation of the current work. Low power creates additional difficulty in interpreting the findings, as it reduces the chance for a true effect to be detected and increases error rates. While the current work shows interesting and important findings and a strong indication for an association between parent stereotypes and bias against parents in hiring contexts, the findings of Study 2 should be interpreted with caution due to the small sample size. Future research could profit from attempting to replicate these findings using a similar paradigm but ensuring a larger sample size.”

“While power in Study 2 of the current work was lower than desirable, the findings provide valuable and novel insight into the processes that may underlie the motherhood penalty effect. These findings highlight an important area for future exploration, focused on the association between caregiver stereotypes and bias against caregivers in career settings.”
